# ﻿Genomics and ecology of *Epibryaceae*, a psychrophilic family in *Chaetothyriales*

**DOI:** 10.3897/imafungus.16.170120

**Published:** 2025-12-22

**Authors:** Bruno Paulo Rodrigues Lustosa, Ricardo Belmonte-Lopes, Sybren de Hoog, Flavia de Fatima Costa, Bruna Jacomel, Germana Davila dos Santos, Emanuel Razzolini, Yalong Li, Ruoning Xue, Valter A. Baura, Emanuel M. de Souza, Renata Rodrigues Gomes, Sarah A. Ahmed, Laura Selbmann, Yinggai Song, Vania Aparecida Vicente

**Affiliations:** 1 Bioprocess Engineering and Biotechnology Graduate Program, Federal University of Paraná, Curitiba, PR, Brazil; 2 Centre of Expertise for Mycology of Radboud University Medical Centre / Canisius Wilhelmina Hospital, Nijmegen, Netherlands; 3 Department of Ecological and Biological Sciences, Tuscia University, Viterbo, Italy; 4 Research Center for Medical Mycology, Peking University, Beijing, China; 5 Microbiology, Parasitology and Pathology Graduate Program, Federal University of Paraná, Curitiba, PR, Brazil; 6 Laboratory of Artificial Intelligence Applied to Bioinformatics and Graduate Program in Bioinformatics, SEPT, Federal University of Paraná, Curitiba, PR, Brazil; 7 Department of Dermatology and Venerology, Peking University First Hospital, Beijing, China; 8 Department of Biochemistry and Molecular Biology, Federal University of Paraná, Curitiba, PR, Brazil; 9 Department of Microbiology, Faculty of Medicine, Kuwait University, Kuwait Kuwait; 10 Italian National Antarctic Museum (MNA), Mycological Section, Genoa, Italy; 11 Microbiological Collections of Paraná Network – Taxonline (CMRP/Taxonline), Federal University of Paraná, Curitiba, PR, Brazil

**Keywords:** Black yeast, comparative genomics, *
Epibryon
*, extremotolerant fungi, gene expansion and contraction

## Abstract

The family *Epibryaceae* is one of the early-diverging lineages within the order *Chaetothyriales*. Available molecular data show that most species are associated with mosses, liverworts, and lichens, typically inhabiting apparently psychrophilic environments. However, genomic information about this family remains scarce. This study presents whole-genome sequencing of six reference strains from the genus *Epibryon* (*Chaetothyriales*, *Epibryaceae*), aiming to elucidate their ecological adaptations and evolutionary relationships. Comparative analyses of CAZymes and MEROPS annotations showed that most members of *Epibryaceae* have a reduced set of enzymes associated with lignin degradation. Additionally, the presence of the CspA protein, linked to freezing tolerance, and the absence of the ClpA/B enzyme, associated with heat stress tolerance, suggest a strong preference for cold environments compared with other *Chaetothyriales* lineages. Multilocus phylogenetic analyses clarified species boundaries and resulted in the introduction of *Epibryon brunneolum* comb. nov. within the family. Based on phylogenetic analysis, ecological data regarding the preferred habitat of the family, and the presence of exclusive enzymes associated with extreme cold environments, the results indicate that this family is distinct from other chaetothyrialean fungi.

## ﻿Introduction

The ascomycete order *Chaetothyriales* comprises melanized fungi closely related to the orders *Verrucariales*, *Pyrenulales*, and *Phaeomoniellales*, which together form the subclass *Chaetothyriomycetidae* within the class *Eurotiomycetes* (Gueidan et al. 2008, 2014; Stenroos et al. 2010; Muggia et al. 2021). Reconstructions of the ancestral state suggest that the order likely originated from melanized, slow-growing, rock-inhabiting fungi (RIF) or lichen-associated ancestors that may have given rise to all extant species within the *Chaetothyriales* (Gueidan et al. 2008, 2011; Muggia et al. 2015, 2021; Quan et al. 2020). Currently, many of these fungi can be found in extreme environments characterized by nutrient scarcity, drastic fluctuations in temperature and hydration, and high levels of UV radiation (Muggia et al. 2021; Coleine et al. 2024). Their ability to thrive under such harsh conditions has led to the recognition of diverse ecological niches, including opportunistic, epiphytic, lichen-associated, rock-inhabiting, ant-associated (e.g., carton and domatia), bryophytic, and toxigenic ecologies (Quan et al. 2020, 2024; Coleine et al. 2024; de Hoog et al. 2024; Wang et al. 2025). These ecological adaptations are thought to be driven by their low competitive ability against co-occurring microorganisms, as evidenced by the need for selective isolation methods to recover these fungi from environmental samples (Vicente et al. 2014, 2017; Costa et al. 2023).

Presently, the order *Chaetothyriales* is divided into at least seven families: *Chaetothyriaceae*, *Cyphellophoraceae*, *Domatiomycetaceae*, *Epibryaceae*, *Herpotrichiellaceae*, *Paracladophialophoraceae*, and *Trichomeriaceae* (Teixeira et al. 2017; Quan et al. 2020, 2024; Wang et al. 2025). Among these, the family *Epibryaceae* is of particular interest, as it represents one of the early-diverging lineages within the order (Gueidan 2014; Döbbeler and Davidson 2019; Fu et al. 2023). The most recognizable genus in this family is *Epibryon*, which comprises over 50 described species mostly isolated from mosses, liverworts, lichens, and rocks (Döbbeler 1978, 1997; Döbbeler and Davidson 2019; Döbbeler et al. 2023). Similarly, other species recently described as *Epibryon* have been isolated from rocks and plants in high-latitude regions and linked to low temperatures (Crous et al. 2007; Davey et al. 2007; Quan et al. 2020; Fu et al. 2023; Kularathnage et al. 2025).

Genomic information on the family *Epibryaceae* remains limited, hindering detailed investigations of their ecology and evolutionary history, especially regarding the elucidation of genes related to their apparently psychrotolerant or psychrophilic tendency (Fu et al. 2023). In this study, whole-genome sequencing (WGS) was conducted for six species of *Epibryaceae* to enable comparisons with other members of *Chaetothyriales* and closely related orders, with the aim of uncovering ecological adaptations and evolutionary relationships.

## ﻿Methods

### ﻿DNA extraction and sequencing

Six reference strains from members of the family *Epibryaceae* were selected for sequencing, including *Epibryon bryophilum* (CBS 126278), *E. humicola* (CBS 117536T), *E. interlamellare* (CBS 126286), *E. minutissimum* (CBS 121758T), *E. turfosorum* (CBS 126587), and *E. sylvestris* (CBS 350.83T). The strains were grown on Sabouraud’s Glucose Agar (SGA) at 28 °C for 7 days. DNA extraction was performed using the cetyltrimethylammonium bromide (CTAB) method with chloroform:isoamyl alcohol (CIA) 24:1 v/v, according to Vicente et al. (2017). DNA concentration was quantified using a Qubit Fluorometer (Invitrogen, Carlsbad, CA, USA), followed by library construction using Nextera XT (Illumina, San Diego, USA) following the manufacturer’s instructions. Sequencing of 150 bp paired-end reads was performed on one of the following platforms: *E. bryophilum*, *E. interlamellare*, and *E. turfosorum* were sequenced with Illumina NovaSeq 6000; *E. humicola* and *E. minutissimum* with Illumina MiSeq 500; and *E. sylvestris* with BGI-Seq500 (MGI, Guangdong, China).

### ﻿De novo assembly, prediction, and annotation

Quality control of the reads was performed using FastQC v0.11.9 (Andrews 2010). Low-quality reads and adapters from the respective sequencing technologies were removed using Trimmomatic v0.39 (Bolger et al. 2014), with a head crop of up to 15 bp when necessary. For assembly of the nuclear genome, the trimmed paired and single reads were subjected to read correction with BayesHammer (Nikolenko et al. 2013) in SPAdes v3.15.5 (Bankevich et al. 2012) and then assembled in SPAdes using the “isolate” mode with k-mer sizes of 31, 51, 71, 91, and 111, followed by gap closing and polishing of the assemblies with GapCloser v1.12 (Luo et al. 2012). The resulting assemblies were filtered to remove contamination from non-eukaryotic DNA using Tiara (Karlicki et al. 2022) with a minimum scaffold length of 500 bp. Additionally, to evaluate the completeness of the assembled genomes, BUSCO analysis was performed using the Chaetothyriales_odb10 trained database (Simão et al. 2015; Kriventseva et al. 2018; Manni et al. 2021).

Gene prediction and annotation were performed using the Funannotate pipeline v1.8.15 (Palmer and Stajich 2022). Initially, the pipeline was used to pre-trim the assembled genomes to remove scaffolds smaller than 500 bp. Subsequently, the scaffolds were sorted by size, and repetitive scaffolds were removed using the “mask” step. Prediction was then performed using the self-training mode of the GeneMark-ES v4.71 program (Lomsadze et al. 2005) with the ab initio predictors Augustus v3.4.0 (Stanke and Morgenstern 2013), SNAP v2006-07-28 (Korf 2004), and GlimmerHMM v3.0.4 (Majoros et al. 2004), trained with the orthologous gene database Chaetothyriales_odb10 (Kriventseva et al. 2018). The predictions from GeneMark-ES and Augustus were combined using Evidence Modeler v1.1.1 (Haas et al. 2008). The predicted genes were then annotated using the following software and databases: (1) InterProScan v5.63-95 (Jones et al. 2014) with the InterPro v95 database; (2) HMMER v3.3.2 (Eddy 2011) with the PFAM v35 (Finn et al. 2013) and dbCAN v11 (Zhang et al. 2018) databases; (3) DIAMOND v2.1.8 (Buchfink et al. 2021) with the MEROPS v11 (Rawlings et al. 2012), UniProt v2023_03 (The UniProt Consortium 2023), Repeats v1.0 (Domenico et al. 2014), and MIBiG v1.4 (Medema et al. 2015) databases; and (4) eggNOG Mapper v2.1.11 (Hernandez-Plaza et al. 2021) on the EggNOG 5.0 database (Huerta-Cepas et al. 2019). Annotation results from the UniProt and eggNOG databases were combined using Gene2Product v1.88 software. All genomes obtained were deposited in the NCBI database (BioProjects PRJNA1129533 and PRJNA1208936).

### ﻿Gene orthology, phylogenomic, and phylogenetic analysis

Evolutionary analysis of the family *Epibryaceae* was performed based on gene orthology, phylogenomic, and phylogenetic data. For this purpose, the assembled genomes from strains of *Epibryaceae* were compared with publicly available reference genomes of species from the orders *Chaetothyriales*, *Phaeomoniellales*, *Pyrenulales*, and *Verrucariales* listed in GenBank (Suppl. material 1: table S1). The genome assemblies were downloaded and subjected to gene prediction and annotation following the methodology described above. Gene orthology analysis was performed using OrthoFinder2 v2.5.5 (Emms and Kelly 2019), focusing on classifying genes according to their presence in different orthogroups (e.g., present in one, two, three, four, or more orthogroups). Phylogenomic analysis was conducted to identify sets of orthologous genes among the analyzed strains using default parameters for DNA sequence analysis. Homology searches were performed with DIAMOND v2.1.13, and the orthogroup sequences were aligned with MAFFT v7.526 (Katoh and Standley 2013). A total of 296 orthogroups, with a minimum of 84.7% of the species having single-copy genes in any orthogroup, were used for ML tree inference with FastTree v2.1.11 (Price et al. 2010).

A multilocus phylogenetic analysis was performed for species delimitation within the family *Epibryaceae* using ribosomal genes (rDNA), including the large subunit ribosomal gene (LSU), small subunit ribosomal gene (SSU), internal transcribed spacer region (ITS), and the RNA polymerase II gene (RPB2) from sequences obtained from reference strains (Suppl. material 1: table S2). Ribosomal genes of the WGS*Epibryaceae* species were extracted from the trimmed paired reads by genome skimming with GetOrganelle v1.7.7.0 (Jin et al. 2020) using the fungi_nr v0.0.1 ribosomal database with up to 20 rounds of read recruitment and multiple k-mer values (21, 45, 65, 85, 105) for the assembly of the recruited reads. Additionally, RPB2 sequences from the genomes were extracted based on the functional annotation using Funannotate v1.8.15. All sequences were aligned using MAFFT v7 (Katoh et al. 2013), and phylogenetic analysis was performed by maximum likelihood with 10,000 replicates using IQ-TREE v2.3.6 (Nguyen et al. 2015) with default parameters.

### ﻿Functional genomic comparison of genes associated with extremotolerance

Genes previously reported in the literature to be associated with the survival of black yeasts in extreme, cold, or hot environments and with UV-radiation tolerance were selected for comparison across all genomes in this study. These include genes identified as up- or downregulated in transcriptomic studies or present in higher copy numbers in chaetothyrialean species occupying distinct ecological niches (Blasi et al. 2014; Wang et al. 2014; Tesei et al. 2015; Jung et al. 2016; Moreno et al. 2017; Teixeira et al. 2017; Vicente et al. 2017; Quan et al. 2024; Coleine et al. 2024). For this comparison, functional PFAM domains and InterPro families of the selected genes were assessed based on annotations generated using the same methodology described above.

### ﻿Gene expansion and contraction

Ancestral state reconstruction based on gene expansion and contraction in the phylogenomic tree was performed using the function “ace” from the ape package v5.0 (Paradis and Schliep 2019) in R (R Core Team 2024). For this analysis, the phylogenomic tree was compared with the copy number of each gene associated with extreme cold and heat stress, generating a separate phylogenetic tree for each gene. In addition, genes annotated from the Carbohydrate-Active enZymes (CAZyme) and MEROPS peptidase databases were included to assess the expansion and contraction of metabolic pathways. The analysis used the “continuous” mode under a Brownian motion (BM) model, which assumes that characters evolve randomly following a random walk (Felsenstein 1973; Schluter et al. 1997). This approach estimated the possible ancestral copy numbers at each node of the phylogenomic tree. In this context, ancestral states were calculated for all nodes of the phylogenomic tree. Afterwards, only genes showing evidence of expansion or contraction at node VI (representing the last common ancestor of the order *Chaetothyriales*) and node VII (representing the last common ancestor of the family *Epibryaceae*) were selected to infer potential expansions and contractions in these lineages. Genes associated with hot and cold stress are shown as separate phylogenetic trees, while the CAZyme and MEROPS gene expansions and contractions at the respective nodes are presented in the Suppl. material 1: table S3.

## ﻿Results

### ﻿Genome assembly and annotation

Whole-genome sequencing (WGS) of six species—*Epibryon bryophilum*, *E. interlamellare*, *E. turfosorum*, *E. sylvestris*, *E. humicola*, and *E. minutissimum*—within the family *Epibryaceae* was performed and compared with other members of the *Chaetothyriales* and closely related orders. De novo assemblies of the new genomes showed an average size of 29.09 Mbp (ranging from 26.20 to 34.97 Mbp). The final annotation revealed an average of 10,495 predicted genes, ranging from 9,908 to 10,915 genes (Table 1).

**Table 1.** *Epibryaceae* genome data assemblies of six species of *Epibryon*.

Species	Strain	Genome size (Mbp)	Number of contigs	GC content (%)	Number of proteins predicted	Number of duplications*	GenBank accession number
*E. bryophilum*	CBS 126278	30.74	303	51.05	10776	38	JBQQWO000000000
*E. humicola*	CBS 117536^T^	27.05	939	49.9	10329	76	JBQQWI000000000
*E. interlamellare*	CBS 126286	26.20	79	50.39	9908	43	JBQQWN000000000
*E. minutissimum*	CBS 121758^T^	27.79	916	49.83	10537	206	JBQQWH000000000
*E. sylvestris*	CBS 350.83^T^	27.76	120	51.16	10506	42	JBQQWE000000000
*E. turfosorum*	CBS 126587	34.97	642	48.96	10915	80	JBQQWM000000000

*Number of duplications was assigned with OrthoFinder v2.5.5 based on 41,386 orthogroups.

Genome completeness estimated with BUSCO using 6,265 genes from the *Chaetothyriales* odb_10 database showed the genomes to have an average of 85.8% complete copies (ranging from 84.2 to 87.2), of which 0.48% (0.7 to 0.3) were duplicated and 85.3% (86.7 to 83.8) were single copies. Additionally, 0.65% (1.0 to 0.5) were considered fragmented, and 13.5% (12.4 to 14.8) were considered missing genes.

The genome comparison using OrthoFinder v2.5.5 was performed for the family *Epibryaceae*, including additional publicly available genomes from the *Chaetothyriomycetidae* subclass, of which one was from the order *Phaeomoniellales*, two from the *Pyrenulales*, two from the *Verrucariales*, and 62 from the *Chaetothyriales*. Of the latter, 36 belonged to the family *Herpotrichiellaceae*, nine to *Trichomeriaceae*, six to *Domatiomycetaceae*, two to *Cyphellophoraceae*, one to *Chaetothyriaceae*, and one genome was from a species identified as incertae sedis. The genome accession numbers are presented in the Suppl. material 1: table S1.

**Figure 1.**
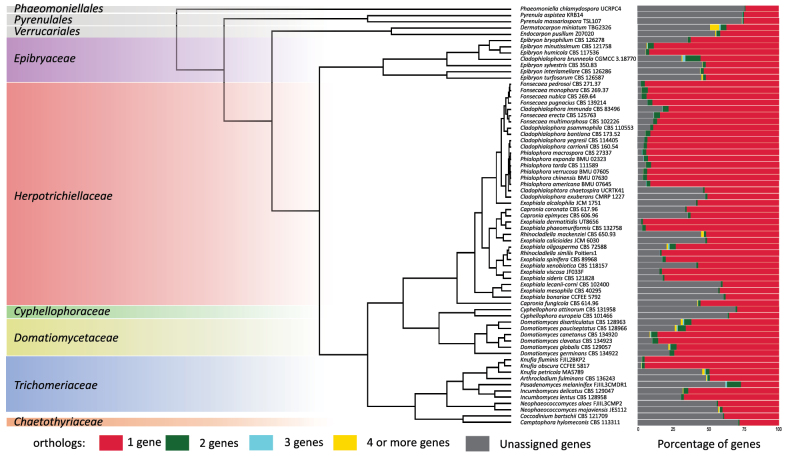
Maximum likelihood phylogenomic tree and core ortholog analysis. The phylogenomic tree was inferred with FastTree v2.1.11 using 296 orthogroups identified with OrthoFinder v2.5.5, each containing single-copy genes in at least 84.7% of the analyzed species. Proportion of genes per species assigned to orthogroups. The bar plot shows, for each genome, the percentage of orthogroups containing a single gene (red), two genes (green), three genes (blue), or four or more genes (yellow) assigned per species. The proportion of genes not assigned to any orthogroup is represented in gray.

Phylogenomic analysis showed that all *Epibryon* species shared a common ancestor and that the species *Cladophialophora brunneola* (CGMCC 3.18770T) is also part of this family (Fig. 1). Ortholog analysis revealed that members of *Epibryaceae* have between 51.8% and 98.3% of their genes assigned to an orthogroup. *Cladophialophora brunneola* CGMCC 3.18770T, isolated from karst rock, when compared with other species originating from moss, presented a higher proportion of orthogroups with two genes assigned (10.7%, Fig. 1, bar plot marked in green).

Regarding the proportion of shared orthogroups within the *Epibryaceae*, *E. humicola* CBS 117536T and *E. minutissimum* CBS 121758T shared 98.3% of orthogroups. In contrast, the remaining strains shared, on average, 50.5% of orthogroups, with values ranging from 34.4% to 71.8% (Suppl. material 1: table S4).

**Figure 2.**
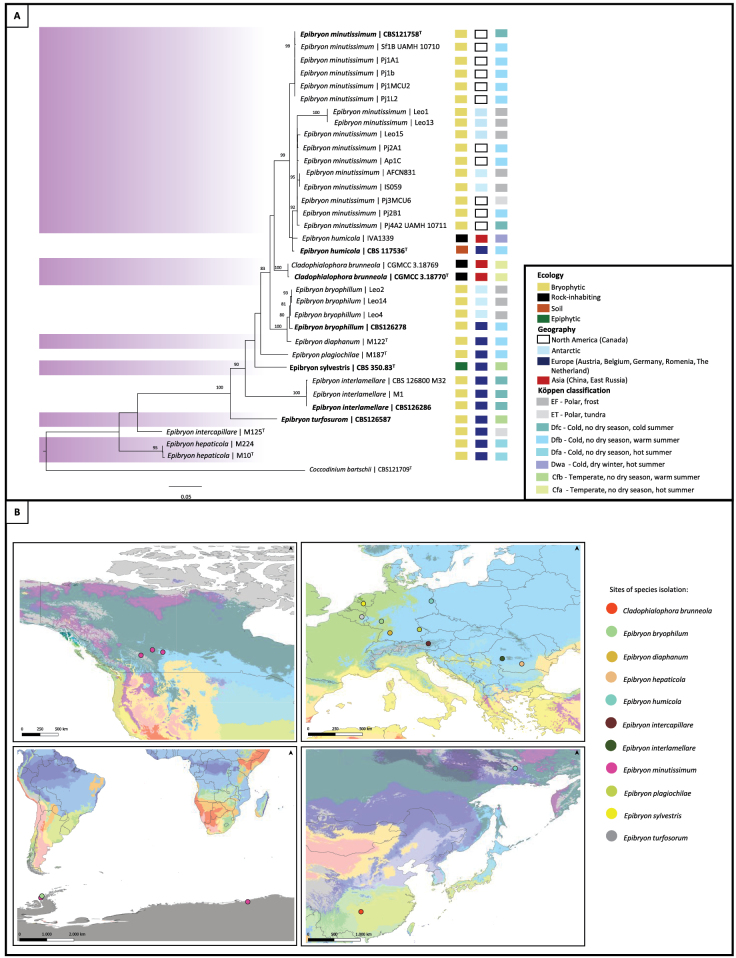
Phylogenetic tree and global distribution of family *Epibryaceae* members. **A** Maximum likelihood phylogenetic tree of the family *Epibryaceae* based on SSU, ITS, LSU, and RPB2 gene sequences inferred with IQ-TREE v2.4.0. Bootstrap values were calculated from 10,000 replicates; values > 80% are shown. The tree was rooted with *Coccodinum bartschii* CBS 121709. Species with sequences extracted from genomes are shown in bold. T = ex-type strains. **B** Geographic distribution of isolation sites for *Epibryaceae* strains. Some locations correspond to more than one isolate. Coordinates for each strain included in the phylogenetic tree are provided in Suppl. material 1: table S2. Circles represent isolated species, with each color indicating a different taxon. Background colors on the map follow the Köppen climate classification (Beck et al. 2023), with the legend shown in Suppl. material 1: fig. S1.

The multilocus analysis (Fig. 2A) based on the ribosomal genes (SSU, ITS, LSU) and RPB2 is concordant with the genome tree (Fig. 1). This phylogenetic analysis revealed that *C. brunneola*, *E. bryophilum*, *E. interlamellare*, *E. sylvestris*, and *E. turfosorum* represent well-supported taxa. In contrast, *E. humicola* and *E. minutissimum* yielded statistically insignificant differences to support them as separate species (Fig. 2A). Additionally, the present analysis indicates that *Cladophialophora brunneola*, previously described as an early-diverging lineage within the *Chaetothyriales* (Fu et al. 2023), is placed within the family *Epibryaceae* (Figs 1, 2A), suggesting that this species is better classified in the genus *Epibryon*. Moreover, *E. hepaticola* and *E. intercapillare* cluster on a separate branch from the main *Epibryon* group (Fig. 2A).

Among isolates with available molecular data, no ecological differences were observed among species of *Epibryaceae* (Suppl. material 1: table S2). Most species were described in association with mosses (Döbbeler 1978, 1979; Davey et al. 2007; Stenroos et al. 2010; Newsham et al. 2014; Hirose et al. 2016), indicating a consistent ecological niche (Fig. 2A). In contrast, some species were reported from rock surfaces (Fu et al. 2023), soil (Crous et al. 2007; Iliushin et al. 2022), or as epiphytes (Crous et al. 2007). Based on Köppen climate classification (Beck et al. 2023), several isolates originated from polar regions as well as cold and temperate continental zones, with precipitation distributed throughout the year and temperatures ranging from extremely cold to humid subtropical (Fig. 2B).

### ﻿CAZyme and MEROPS in the family *Epibryaceae*

**Figure 3.**
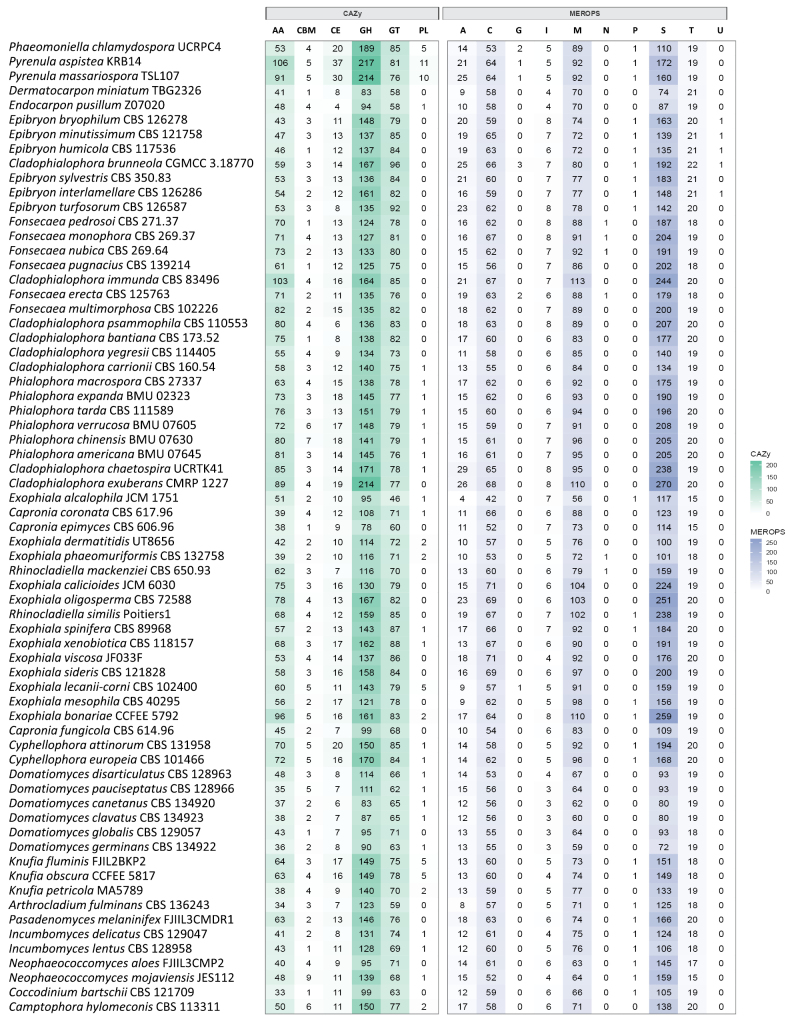
Heatmap of CAZyme and MEROPS families according to genome annotation. Copy numbers of each enzyme family are shown in the squares.

On average, species within the family *Epibryaceae* had 297 genes assigned to the Carbohydrate-Active enZymes (CAZyme) database, with values ranging from 280 to 339, encompassing a total of 106 different CAZyme families (Suppl. material 1: table S5). There was an average of 50.7 auxiliary activity (AA) enzymes (range: 46–59), 2.5 carbohydrate-binding modules (CBM; range: 1–3), 11.8 carbohydrate esterases (CE; range: 8–14), 145.9 glycoside hydrolases (GH; range: 135–167), and 86.0 glycosyltransferases (GT; range: 79–96), with no genes assigned to the polysaccharide lyase (PL) family (Suppl. material 1). *Cladophialophora brunneola* possessed the highest number of CAZyme genes across all *Epibryaceae*. In contrast, *E. bryophilum* had the lowest copy numbers of AA and GT genes; *E. humicola* had the fewest CBM genes; and *E. turfosorum* showed the lowest counts of CE and GH genes among the analyzed species (Fig. 3).

Analyses of MEROPS proteases showed that members of the *Epibryaceae* had an average of 345.7 genes annotated in this database, with values ranging from 318 to 397 genes, representing 89 distinct MEROPS families (Suppl. material 1: table S6). Regarding distribution across different peptidase classes, the genomes contained an average of 20.4 genes belonging to the aspartic (A) family (range: 16–25), 62.0 to the cysteine (C) family (range: 59–66), 0.42 to the glutamic (G) family (range: 0–3), 7.1 to the inhibitor (I) family (range: 6–8), 75.7 to the metallo (M) family (range: 72–80), one to the mixed (P) family, 157.4 to the serine (S) family (range: 135–192), 20.8 to the threonine (T) family (range: 20–22), and 0.7 to the unknown (U) family (range: 0–1), with no copies assigned to the asparagine (N) family. Within this context, *C. brunneola* exhibited the highest number of genes from the A, C, M, S, and T families and was the only species with genes assigned to the G family, with three copies. In contrast, *E. bryophilum* and *E. turfosorum* contained the highest number of genes assigned to the I family, each with eight copies. *E. interlamellare* had the fewest genes from the A family, while both *E. interlamellare* and *E. bryophilum* showed the lowest counts for the C family. *E. minutissimum* and *E. humicola* had the lowest counts for the I, M, and S families, and both *E. bryophilum* and *E. turfosorum* had the fewest genes in the T protease family (Fig. 3).

### ﻿Expansion or contraction of CAZyme and MEROPS

The analysis of CAZyme and MEROPS gene copy numbers revealed multiple expansions and losses within the *Chaetothyriales* lineage compared with the outgroups. CAZyme families AA1, AA4, GH1, GH3, GH10, GH13, GH16, GH20, GH27, GH28, GH31, GH76, GT22, and GT76 showed clear signatures of expansion in the last common ancestor of the order *Chaetothyriales* (Suppl. material 1: table S3). For example, AA1 (laccases) increased from approximately seven copies in the ancestral state to 11–20 copies across the order, and GH3 (glucosidases) expanded from six copies to an average of 8–13. In addition, several gene families originated and expanded in certain species within the order, including AA14, CBM13, CBM52, CE2, CE3, GH10, GH12, GH28, GH29, GH33, GH39, GH105, GH115, GH127, GH154, GH162, GT76, GT91, and GT109. Conversely, some contractions were observed compared with the ancestral state, such as a reduction in GH114 (Suppl. material 1: table S3).

MEROPS peptidase analysis also indicated expansion within the *Chaetothyriales* profile. The ancestral state of this order showed expansion of proteins A01A, M20D, M38, S10, S12, S28, S33, and S09X—the latter (a prolyl oligopeptidase) increasing from approximately 26 copies in the ancestral state to 37–87 copies across families. Similarly, MEROPS families such as N09, S09A, and U74 were inferred to have originated within the order. In contrast, contractions or complete losses were observed for families C85, M04, M13, M35, and M77, which are absent in several lineages relative to the ancestral *Chaetothyriales* (Suppl. material 1: table S3).

Within the family *Epibryaceae*, a distinct CAZyme and MEROPS profile was observed compared with other genomes. For instance, the *Epibryaceae* possess the exclusive CAZyme CBM52, and the ancestral lineage of the family showed expansions of AA5 and GH29—the latter being shared only with the *Domatiomycetaceae*. In addition, GT25 and GT34 were expanded compared with other families, while AA7, AA9, and GH12 showed contractions within the *Epibryaceae* (Suppl. material 1: table S3). Regarding MEROPS, the *Epibryaceae* ancestral state showed expansions in S53 and T02 compared with other families, and members of this family uniquely possess the enzyme U74, which has a nonspecific catalytic site capable of digesting proteins without substrate size restriction (Rey et al. 2016; Ting et al. 2022). Meanwhile, contractions were observed for C26 and C85 proteases (Suppl. material 1: table S3).

### ﻿Expansion or contraction of genes associated with heat and cold stress tolerance and UV-radiation tolerance

**Figure 4.**
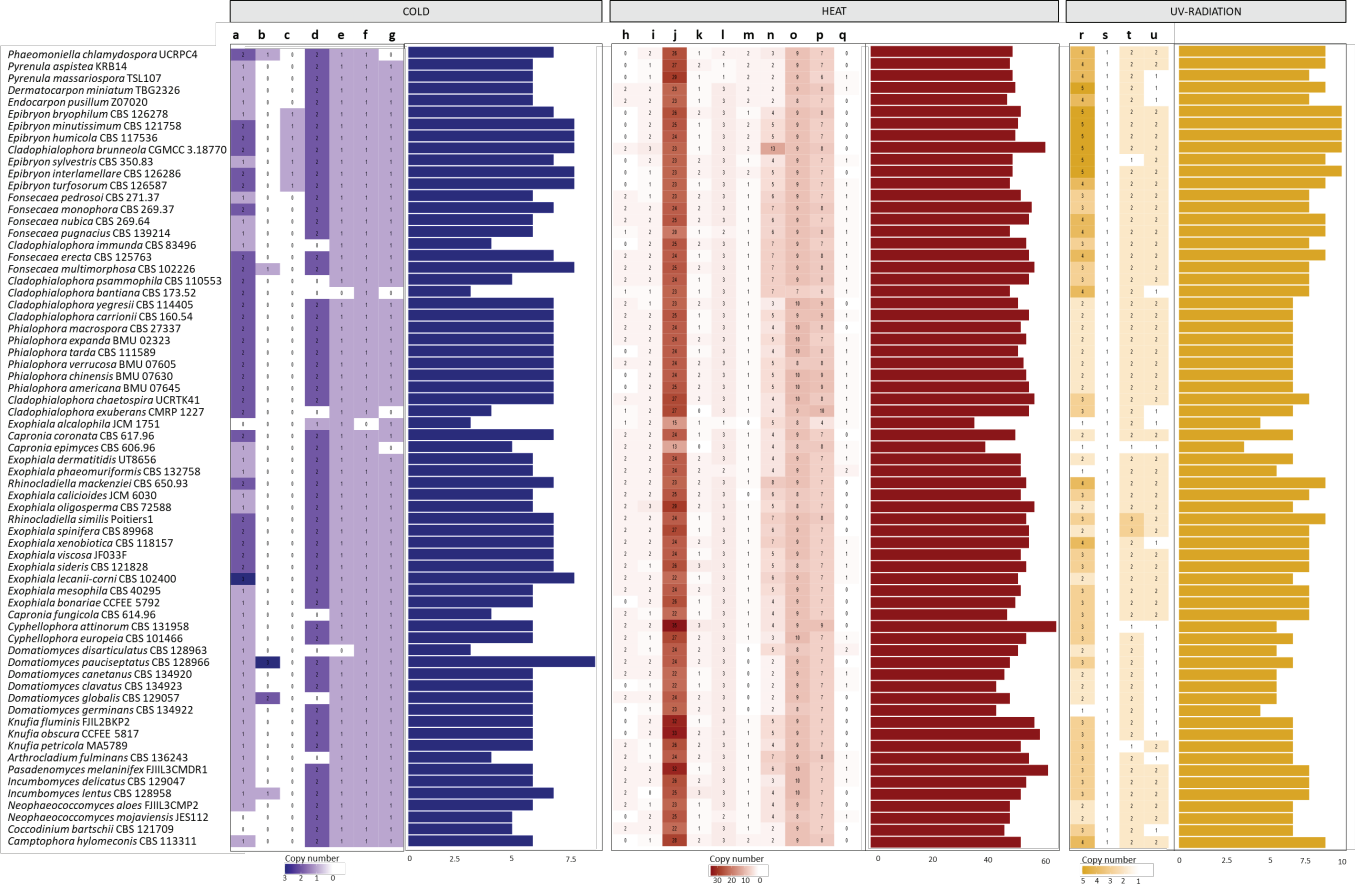
Heatmap and total number of heat- and cold stress-associated proteins. Copy numbers of enzymes are shown in the squares. Total numbers of proteins associated with heat and cold stress tolerance are shown as bar plots. Proteins associated with cold stress tolerance: a = 5-formyltetrahydrofolate cyclo-ligase (PF01812/IPR002698), b = antifreeze protein, type I (IPR000104), c = CspA; cold shock protein (IPR012156), d = dihydrolipoamide acetyltransferase (IPR045257), e = electron transfer flavoprotein alpha subunit (IPR001308), f = low-temperature viability protein (PF04180), g = small subunit ribosomal protein S16 (IPR023803). Proteins associated with heat stress tolerance: h = ClpA/B, conserved site 2 (IPR028299), i = CSL zinc finger (PF05207), j = DnaJ (PF00226), k = enolase (IPR000941), l = Hsf1_family (PF00447/IPR000232), m = HSP12 (PF04119), n = HSP20 (PF00011), o = HSP60 (PF00118), p = HSP70 (PF00012), q = HSP90 (PF00183, PF13589, PF02518), r = DNA recombination/repair protein Rad51 (IPR011941), s = DNA repair protein RAD54 with RDH54 (PF08658), t = universal stress protein (PF00582, PF10625), u = UV-radiation resistance-associated protein Vps38/UVRAG (PF10186, PF17649).

Annotation of genes associated with heat stress tolerance revealed that members of the *Chaetothyriales* share a diversity of ten different enzymes. Genes linked to cold stress tolerance showed seven distinct enzymes within the order, while genes associated with UV-radiation tolerance included four predicted enzymes (Fig. 4).

The family *Epibryaceae* possesses an average of 54.0 copies of enzymes related to heat stress tolerance (range: 50–62), similar to other families within the *Chaetothyriales*. However, the *Epibryaceae* exhibit an average of 7.7 copies of enzymes associated with cold stress tolerance (range: 7–8), representing an increase compared with other families in the order. For genes associated with UV-radiation tolerance, the *Epibryaceae* show an average of 9.7 gene copies (range: 9–10), whereas other *Chaetothyriales* families display an average of 7.3 (Fig. 4).

**Figure 5.**
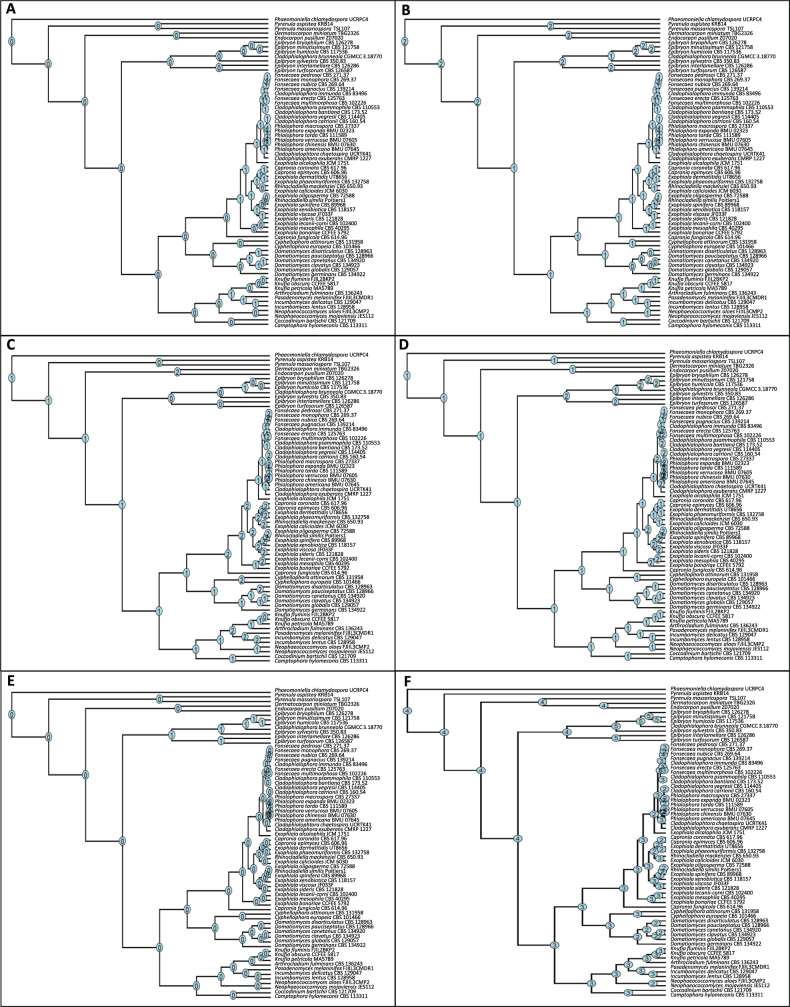
Phylogenomic trees showing expansion and contraction in the copy number of enzymes associated with heat and cold stress. **A** HSP90 (PF00183, PF13589, PF02518); **B** HSP12 (PF04119); **C** ClpA/B, conserved site 2 (IPR028299); **D** 5-formyltetrahydrofolate cyclo-ligase (PF01812/IPR002698); **E** cold shock protein CspA (IPR012156); **F** DNA recombination/repair protein Rad51 (IPR011941).

The expansion and contraction analysis of heat stress-related genes showed an expansion of the gene HSP90 (PF00183, PF13589, PF02518), which was absent in the ancestral node but present in some species of the *Herpotrichiellaceae* and *Domatiomycetaceae* (Fig. 5A). In contrast, a loss of one copy of the enzyme HSP12 (PF04119) was observed in the ancestor of the *Herpotrichiellaceae* (Fig. 5B). Within the *Epibryaceae*, the enzyme ClpA/B, conserved site 2 (IPR028299), was absent (Fig. 5C).

Regarding cold stress-related genes, the analysis revealed a duplication of the enzyme 5-formyltetrahydrofolate cyclo-ligase (PF01812/IPR002698) in the last common ancestor of the *Epibryaceae* (Fig. 5D). Additionally, the *Epibryaceae* is the only family in the order that harbors the cold shock protein CspA (IPR012156) (Fig. 5E).

In relation to genes associated with UV-radiation tolerance, there was an expansion in the DNA recombination/repair protein Rad51 (IPR011941). The last common ancestor of the *Chaetothyriales* had four copies, whereas members of the *Epibryaceae* have five copies (Fig. 5F). Moreover, several members of the *Herpotrichiellaceae* possess only two copies.

## ﻿Discussion

The present study compared the functional genomic profile of various *Epibryon* species with other members of the order *Chaetothyriales* and with closely related taxa in other orders of the subclass *Chaetothyriomycetidae*. The phylogenomic results support previous studies in which *Epibryon* was recognized as a distinct family, the *Epibryaceae*, first described by Stenroos and Gueidan (2014, MB#808432). Members of this family are mostly biotrophic or saprobiotic on foliose hepatics and mosses that grow on the older, dying parts of the lamella (Döbbeler 1978, 1979; Döbbeler et al. 2023). Currently, *Epibryon* comprises about 50 species (Index Fungorum, May 2025), which are morphologically characterized by small, setose ascomata 40–150 µm in diameter, perithecioid, globose to ovoid or pyriform, ostiolate, and light to dark brown or black, with straight or curved dark setae (Döbbeler 1978; Gueidan 2014). Sequence data available for some of these species have placed them in an ancestral position within the *Chaetothyriales* (Quan et al. 2020). Three *Cladophialophora* species (*C. humicola*, *C. minutissima*, and *C. sylvestris*)—named for their long, coherent conidial chains produced in culture—were found to cluster within this group, distant from the generic type species *C. carrionii*, a thermophilic human pathogen belonging to the *Herpotrichiellaceae*. These species have now been reassigned to *Epibryon* (Kularathnage et al. 2025). Additionally, our data indicate that another catenate fungus, *Cladophialophora brunneola*, also clusters within the *Epibryaceae*. Its ecology as a rock-inhabiting fungus isolated at 2,220 m altitude from a karst landform in Guizhou, China (Fu et al. 2023), matches the lifestyle of *Epibryon* species. For this reason, the following reclassification is proposed:

### ﻿ Epibryon brunneolum

Taxon classificationAnimalia

(W. Sun, Lei Su, M.C. Xiang and Xing Z. Liu) B.P.R. Lustosa, S. de Hoog, V.A. Vicente and Y. Song comb. nov.

7323A90C-9503-54EE-9DD2-E9998310184E

860038

#### Basionym.

*Cladophialophora brunneola* W. Sun, Lei Su, M.C. Xiang and Xing Z. Liu – Mycology 14: 330, 2023.

*E. brunneolum* genome exhibited the highest proportion of orthogroups with two genes compared with other *Epibryaceae* species (Fig. 1), showing greater protein diversity. Previous studies have shown that this species also has the highest number of CAZymes among rock-inhabiting *Chaetothyriales* (Fu et al. 2023). Rock-inhabiting fungi (RIF) experience severe fluctuations in temperature and humidity as they occupy sun-exposed rock surfaces (Quan et al. 2024). In this context, recent studies have demonstrated that RIF can exhibit expansions in cell wall biosynthesis, lipid metabolism, and stress-response pathways (Fu et al. 2025), which may explain their adaptation to such specialized niches.

In addition to mosses and hepatics, previous studies have reported several *Epibryaceae* isolates from lichens (Muggia et al. 2015). In the derived chaetothyrialean family *Herpotrichiellaceae*, heat tolerance is often associated with potential pathogenicity in humans, as seen in chromoblastomycosis caused by *Cladophialophora carrionii* (Deng et al. 2013). Moreover, associations between *Cladophialophora* species and lichens have been documented (Chang et al. 2023; Cometto et al. 2023; Costa et al. 2025; Keller et al. 2025). Related taxa such as *Melanina*, *Muellerella*, and *Paracladophialophora*—rock- and lichen-inhabiting fungi of uncertain phylogenetic placement within *Chaetothyriales*—show a similar affinity for lichenized habitats (Muggia et al. 2015, 2021; Cometto et al. 2023; Quan et al. 2024). These findings suggest that lichen symbiosis is not uncommon within the order. Expanded phylogenetic analyses incorporating newly described taxa, such as *Pleostigma*, have also revealed multiple lichen-associated species positioned between the *Verrucariales* and *Chaetothyriales*, indicating that lichen association may represent an ancestral state of both orders (Muggia et al. 2015, 2021; Costa et al. 2025).

Compared with other members of the *Chaetothyriales*, the *Epibryaceae* exhibit a distinct repertoire of enzymes related to cold stress tolerance (Fig. 4), as well as CAZymes and MEROPS peptidases (Fig. 3). The expansion of specific cold-related genes suggests that members of this family are well adapted to extremely low-temperature environments. All *Epibryaceae* species possess the cold shock protein A (CspA), an enzyme with RNA-binding activity that stabilizes ribosomal structures for protein translation under cold stress (Fang and Leger 2010). Previous work has shown that this enzyme is expanded among black fungi in the class *Dothideomycetes* isolated from Antarctic environments (Coleine et al. 2024), indicating its key role in fungal survival at low temperatures. The absence of ClpA/B, conserved site 2 (IPR028299), in *Epibryon* species suggests a reduced tolerance to high temperatures, as this domain is known to protect cellular structures during heat stress (Barnett et al. 2000; Jaworek et al. 2014). Although its function in fungi remains poorly understood, studies indicate its importance for heat stress tolerance. Furthermore, the expansion of DNA recombination/repair protein Rad51 (IPR011941) observed in all members of *Epibryaceae* may contribute to UV radiation tolerance, as this gene encodes a protein involved in repairing DNA damage caused by UV light (Jung et al. 2016). This gene is also expanded in Antarctic black fungi of *Dothideomycetes* (Coleine et al. 2024), highlighting its potential role in resistance to high UV radiation in glacial environments. Together, these adaptations may explain the preference of *Epibryaceae* for psychrophilic habitats compared with other *Chaetothyriales* lineages.

The *Chaetothyriales* show expansions in several gene families, including AA1 (laccase), AA4 (vanillyl-alcohol oxidase), and S09X (carboxylesterase), as reported previously (Teixeira et al. 2017; Vicente et al. 2017; Moreno et al. 2017). The expansion of various CAZyme glycoside hydrolase families suggests that *Chaetothyriales* species possess numerous enzymes involved in plant biomass degradation compared with their ancestral group (Suppl. material 1: table S3). This supports the hypothesis that these fungi may have originated in rock- and lichen-associated habitats and later evolved toward a saprobic lifestyle (Gueidan et al. 2014; Quan et al. 2024). Although the *Epibryaceae* display a similar pattern of gene family expansions, they also show contractions in certain auxiliary activity families, such as AA7 (glucooligosaccharide oxidases), AA9 (monooxygenases), and GH12 (glucanases) (Suppl. material 1: table S3). Additionally, members of the family possess the exclusive CBM52—binding to endo-1,3-β-glucanase—an auxiliary enzyme that enhances β-glucanase activity in cell septation (Martín-Cuadrado et al. 2008). The specific role of this domain in the ecological adaptation of *Epibryon* remains to be elucidated. The reduced set of lignin- and polysaccharide-degrading enzymes, along with contraction in cysteine peptidases involved in thiol group hydrolysis (Shestakova et al. 2024), indicates a lower overall peptidolytic and ligninolytic capacity. These features likely contribute to the family’s preference for moss-associated habitats in cold environments, where lignin-rich plant material is less abundant and easily accessible carbon sources are supplied through a saprobiotic relationship (Gueidan et al. 2008; Stenroos et al. 2010; Muggia and Grube 2018; Costa et al. 2025).

The psychrophilic preference of *Epibryon* distinguishes it from other chaetothyrialean fungi and aligns it with lichenicolous fungi that inhabit rocks in boreal and Arctic climates. Its saprobiotic ecology with mosses and lichens suggests an atypical niche within the *Chaetothyriales*, as such ecology is rare in this order. *Ascomycetes* that are obligate bryophyte saprobes typically inhabit microsites such as leaf nerves or hyaline hair points in mosses, subterranean rhizoids, leaf borders or axils, and even individual leaf cells in foliose hepatics (Döbbeler et al. 2023). Most microniches described for *Epibryon* occur on the lower, dying, or dead leaves of various bryophyte species. Some species appear to exhibit host specificity (Döbbeler 1978, 1979, 1997; Döbbeler et al. 2023). However, despite extensive herbarium records, molecularly characterized isolates remain scarce (Stenroos 2010), limiting understanding of their ecological distribution. Our data indicate that the habitat preference of *Epibryon* species is broader, also encompassing lichens, soil, and the phyllosphere. Future sampling and molecular sequencing of *Epibryon* from underexplored regions will further clarify its apparent specialization for saprobic lifestyles on mosses, liverworts, and lichens in cold climates.

## ﻿Conclusion

This study expands the scope of genomic comparisons within the subclass *Chaetothyriomycetidae*. The family *Epibryaceae* possesses a distinct set of enzymes associated with cold tolerance, such as the cold shock protein A (CspA), which is absent in other members of the order *Chaetothyriales* (Fig. 5). This supports the hypothesis that the family predominantly inhabits low-temperature environments. Sampling records further confirm that most species with available molecular data have been isolated from cold habitats, including polar regions and mountainous areas (Fig. 2B). Comparative analyses of CAZyme and MEROPS annotations revealed that, while many *Chaetothyriales* exhibit expansions in lignin-degrading enzymes, such as auxiliary activity enzymes, the *Epibryaceae* display a reduced number of these proteins (Suppl. material 1: table S3). This limited enzymatic capacity for lignin and complex polysaccharide breakdown aligns with their association with mosses and lichens, which provide readily accessible carbon sources in nutrient-poor, cold environments. Phylogenomic analyses position the *Epibryaceae* basally within the *Chaetothyriales* and support the inclusion of *Epibryon brunneolum* comb. nov. in the family (Fig. 1). Collectively, the results indicate that the *Epibryaceae* represent a psychrotolerant lineage adapted to cold environments, often in association with mosses and occasionally with lichens, as well as in epiphytic, rock-inhabiting, and soil contexts. These findings suggest that the family shares an ancestral ecological state with members of closely related orders but has evolved unique traits distinguishing it from other *Chaetothyriales*.

## Supplementary Material

XML Treatment for Epibryon brunneolum

## ﻿Additional information

### Use of AI

No use of AI was reported.

### Adherence to national and international regulations

All the fungal strains used in this study have been legally obtained, respecting the Convention on Biological Diversity (Rio Convention).

### Author ORCIDs

Bruno Paulo Rodrigues Lustosa https://orcid.org/0000-0002-0150-5962

Ricardo Belmonte-Lopes https://orcid.org/0000-0002-3122-0271

Sybren de Hoog https://orcid.org/0000-0002-5344-257X

Flavia de Fatima Costa https://orcid.org/0000-0003-4092-4673

Bruna Jacomel https://orcid.org/0000-0001-9552-9369

Germana Davila dos Santos https://orcid.org/0000-0002-0774-354X

Emanuel Razzolini https://orcid.org/0000-0002-9391-5271

Yalong Li https://orcid.org/0009-0006-6914-2877

Ruoning Xue https://orcid.org/0000-0002-4218-5387

Valter A. Baura https://orcid.org/0000-0003-2827-6239

Emanuel M. de Souza https://orcid.org/0000-0003-1546-9218

Renata Rodrigues Gomes https://orcid.org/0000-0002-1827-3408

Sarah A. Ahmed https://orcid.org/0000-0003-0155-8113

Laura Selbmann https://orcid.org/0000-0002-8967-3329

Yinggai Song https://orcid.org/0000-0002-1644-6942

Vania Aparecida Vicente https://orcid.org/0000-0002-2953-4861

### Data availability

All gene and genome sequences generated in this study are available in GenBank under the accession numbers provided. All data supporting the findings of this study are included in the main text and the Supplementary Information.

## Supplementary materials

Supplementary material 1 Additional information Data type xlsx Explanation note **table S1.** Genome accesion public available genomes accessed in the NCBI. **table S2.** Additional information about the reference strains and isolates, coordinates of isolate genes and ecology. **table S3.** Genes expansion and contraction based on copy number of Carbohydrate-Active enZymes. **table S4.** Orthogroups species overlap. **table S5.** Distribution of CAZyme enzymes according to genome prediction from the selected genomes. **table S6.** Distribution of MEROPS enzymes according to genome prediction from the selected genomes. **fig. S1.** Climate plot map according to Köppen-Geiger climate classification. The colors represent a different climate with different seasons. https://binary.pensoft.net/file/1496896This dataset is made available under the Open Database License (http://opendatacommons.org/licenses/odbl/1.0/). The Open Database License (ODbL) is a license agreement intended to allow users to freely share, modify, and use this Dataset while maintaining this same freedom for others, provided that the original source and author(s) are credited.Bruno Paulo Rodrigues Lustosa, Ricardo Belmonte-Lopes, Sybren de Hoog, Flavia de Fatima Costa, Bruna Jacomel, Germana Davila dos Santos, Emanuel Razzolini, Yalong Li, Ruoning Xue, Valter A. Baura, Emanuel M. de Souza, Renata Rodrigues Gomes, Sarah A. Ahmed, Laura Selbmann, Yinggai Song, Vania Aparecida Vicente
